# Standing Crop, Turnover, and Production Dynamics of *Macrocystis pyrifera* and Understory Species *Hedophyllum nigripes* and *Neoagarum fimbriatum* in High Latitude Giant Kelp Forests

**DOI:** 10.1111/jpy.13291

**Published:** 2022-11-17

**Authors:** Lauren E. Bell, Kristy J. Kroeker

**Affiliations:** ^1^ Ecology and Evolutionary Biology University of California Santa Cruz 130 McAllister Way Santa Cruz California 95060 USA

**Keywords:** blue carbon, carbon cycling, carbon dioxide, carbon sequestration, fringe habitat, nitrogen, nutrients, primary productivity, seaweed

## Abstract

Production rates reported for canopy‐forming kelps have highlighted the potential contributions of these foundational macroalgal species to carbon cycling and sequestration on a globally relevant scale. Yet, the production dynamics of many kelp species remain poorly resolved. For example, productivity estimates for the widely distributed giant kelp *Macrocystis pyrifera* are based on a few studies from the center of this species' range. To address this geospatial bias, we surveyed giant kelp beds in their high latitude fringe habitat in southeast Alaska to quantify foliar standing crop, growth and loss rates, and productivity of *M. pyrifera* and co‐occurring understory kelps *Hedophyllum nigripes* and *Neoagarum fimbriatum*. We found that giant kelp beds at the poleward edge of their range produce ~150 g C · m^−2^ · year^−1^ from a standing biomass that turns over an estimated 2.1 times per year, substantially lower rates than have been observed at lower latitudes. Although the productivity of high latitude *M. pyrifera* dwarfs production by associated understory kelps in both winter and summer seasons, phenological differences in growth and relative carbon and nitrogen content among the three kelp species suggests their complementary value as nutritional resources to consumers. This work represents the highest latitude consideration of *M. pyrifera* forest production to date, providing a valuable quantification of kelp carbon cycling in this highly seasonal environment.

**Abbreviation**
FSC: foliar standing crop

Increasing anthropogenic carbon emissions have sharpened the worldwide focus on natural carbon sinks (de Coninck et al. 2018, Lecocq et al. 2022). Terrestrial woody forests and vegetated coastal habitats (mangroves, seagrass meadows, salt marshes) have received the majority of attention due to their capacity to sequester carbon through standing crop and burial. More recently, marine macroalgal (seaweed) ecosystems have been highlighted for their potential as a substantial, climate‐relevant carbon sink (e.g., Krause‐Jensen and Duarte 2016, Laurens et al. 2020, Duarte et al., 2022). Seaweeds – and canopy forming kelp forests in particular – may contribute substantially to “blue carbon” storage through their rapid growth, large standing crop, and the allochthonous burial of their detrital export (Ortega et al. 2019, Queirós et al. 2019, Filbee‐Dexter and Wernberg 2020, Smale et al. 2021). However, the paucity of quality data on kelp production rates (as g Carbon · m^−2^ · year^−1^) is cited as one of the major obstacles to practical estimates of kelp's carbon sequestration capacity (Reed and Brzezinski 2009, Krause‐Jensen et al. 2018).

Research on kelp production is difficult because of the intensive sampling necessary to capture growth and turnover dynamics (Hurd et al. 2014, Schiel and Foster 2015). Production rates of the giant kelp *Macrocystis pyrifera*, a globally abundant and high biomass macroalga, have primarily come from well‐studied regions in the center of this species' latitudinal range (Pessarrodona et al. 2022). Much less is known about carbon cycling within *M. pyrifera* in fringe habitats, particularly at its polar extents (but see Wheeler and Druehl 1986, van Tussenbroek 1989, Attwood et al. 1991). Understanding kelp production capacity in these habitats is needed to correct the geospatial bias of the data that currently inform global estimates of seaweed productivity. High latitude environments are seasonally dynamic, where variation in seawater temperature, *p*CO_2_, storms, light, and nutrient availability impact macroalgal physiology (Graham et al. 2007, Kroeker et al. 2021). The long photoperiod of the high latitude spring and summer contributes to seasonally high rates of production for some macroalgal species (Druehl and Wheeler 1986, van Tussenbroek 1989, Brown et al. 1997, Nielsen et al. 2014). Yet, the duration and magnitude of this production may be constrained by intense competition for nutrients and light with phytoplankton blooms and other macroalgae (Kavanaugh et al. 2009, Miller et al. 2011, Pfister et al. 2019, Bell et al. 2022). Additionally, the physiological tolerance of *M. pyrifera* is likely challenged at certain times of the year near the edge of the range, which may also restrict its overall productivity (e.g., King et al. 2020). Year‐round measurements of giant kelp growth, loss, and foliar standing crop (FSC) from higher latitude regions are therefore necessary to understand the carbon production associated with *M. pyrifera*‐dominated ecosystems worldwide.

While surface‐canopy forming kelp genera (e.g., *Macrocystis*, *Nereocystis*, *Ecklonia*, *Laminaria*) have received the most attention for their carbon production potential, these kelps frequently co‐occur with substantial macroalgal subcanopies. Total production by understory algae has been estimated to rival production by *M. pyrifera* in kelp forests within the center of its range, and it can increase to compensate for production lost if the surface canopy is removed (Miller et al. 2011, Castorani et al. 2021). In higher‐latitude giant kelp forests, understory algal communities are often dominated in biomass by a few species of large, fast‐growing stipitate kelps, such as *Hedophyllum nigripes*, *Neoagarum fimbriatum*, *Agarum clathratum* in the north Pacific (Schiel and Foster 2015, Kroeker et al. 2021). Some of these subcanopy kelp species may have more poleward distributions than the primary canopy forming species (Wulff et al. 2009, Grant et al. 2020), which could influence their relative production capacity in these regions. Studies resolving the comparative ecological performance of subcanopy versus canopy kelps in these regions of overlapping range distributions will provide valuable context ahead of anticipated environmental and species distribution changes (Krumhansl et al. 2016).

In addition to understanding the relative production capacity of different kelp species, investigations of the temporal nature of this relationship in seasonally driven ecosystems may be essential for predicting their vulnerability to global change. As ocean acidification and warming overlay onto current environmental variability at high latitudes, the responses of marine producers may vary by season (Graiff et al. 2015, Wahl et al. 2020). Further, consumers in these systems will experience heightened susceptibility to stressful conditions in particular seasons (Kroeker et al. 2021). Ecological theory suggests that the resilience of these ecosystems will hinge on both the abundance and diversity of basal production that is available to support consumers under such enhanced stress (Bernhardt and Leslie 2013, Gaylord et al. 2015, Doubleday et al. 2019). Although some seasonal complementarity in macroalgal production may already occur due to natural variation in different species' growth phenologies, such fundamental knowledge is still lacking for many high latitude environments. Yet, we know that it is very likely that coexisting macroalgal species will be differentially affected by global change stressors (Phelps et al. 2017, Pessarrodona et al. 2019). Therefore, to predict how future changes could alter the temporal availability of basal energy resources in high latitude coastal marine ecosystems, it is essential that we first understand the current seasonal timing of production for the dominant macroalgal species of the region.

In this study, we provide a novel, multi‐year time series of canopy and subcanopy kelp production in a seasonally dynamic high latitude system. Our research focuses on *Macrocystis pyrifera* beds in Sitka Sound, southeast Alaska, near the northernmost continuous edge of this species' range (Druehl 1970, 1981). Similar to other high latitude regions, temporal variation in the FSC of kelp beds in southeast Alaska is expected to be driven by seasonal variation in temperature, nutrient supply, disturbance from winter storms, and irradiance (Calvin and Ellis 1981, Druehl and Wheeler 1986, Stekoll 2019). However, observations from outer coast areas such as Sitka Sound are sparse. In our study of three of the most common subtidal kelps of this region (*M. pyrifera*, *Hedophyllum nigripes*, *Neoagarum fimbriatum*), we expect to see increased growth and FSC of all species in late winter and early spring, followed by substantial FSC declines in fall and early winter due to physical stress from storm swell. We previously observed that *M. pyrifera* canopies in Sitka Sound begin to degrade and foul by mid‐summer (~July), perhaps due to low nutrient concentrations during this period (Brown et al. 1997, Rodriguez et al. 2016). To investigate the local relationship between kelp production and nutrient supply, we also conduct year‐round sampling to capture the temporal availability of seawater nitrogen in Sitka Sound. In other systems exhibiting seasonal trends in nutrient availability, the nitrogen content of kelps is observed to generally mirror temporal patterns in seawater inorganic nitrogen supply (Wheeler and Srivastava 1984, Brzezinksi et al. 2013, Stephens and Hepburn 2016) though bulk seawater samples do not necessarily reflect all nitrogen sources or supply available to algal tissues (Hurd et al. 1994). Therefore, we also sample a variety of kelp tissues to determine temporal and spatial variability in thallus carbon and nitrogen concentrations. Finally, two of our three sites undergo a phase shift from lush kelp forest to urchin barrens during our study, ostensibly caused by changes in top‐down control (Raymond et al. 2019, Gorra et al. 2022). These unexpected changes facilitate observations of how growth, loss, and production rates of three dominant, interacting kelp species respond to declines in their FSC associated with enhanced grazing pressure.

## METHODS

### Plant biomass and foliar standing crop

We conducted monthly surveys of *Macrocystis pyrifera* for FSC estimation in Sitka Sound, Alaska from January 2017 to February 2018 at Breast Is. (57.039 N, 135.333 W) and Harris Is. (57.032 N, 135.277 W), and seasonally in July 2018, January 2019, and July 2019 at Breast Is., Harris Is., and Samsing Pinnacle (56.988 N, 135.357 W). We surveyed all unique *M. pyrifera* sporophytes (hereafter, “plants”; Bolton 2016) within two permanent 30 × 2 m transects at the 5–7 m depth (MLLW) contour and counted the total number of fronds extending >1 m above the holdfast (hereafter, “frond density”). To determine the relationship between frond density and total wet mass (g), we collected and measured *M. pyrifera* plants (excluding their holdfasts) in summer 2017 (*N* = 16) and winter 2018 (*N* = 10; Kroeker et al. 2021). We used a linear model to test the effect of season (winter and summer) on the relationship between frond density and wet mass. *Macrocystis pyrifera* frond density explained 94% of the variability in total plant wet mass (g) excluding its holdfast, regardless of season (*P* = 0.594; see Table S1 in the Supporting Information for all regression parameter results). In January 2022, we collected *M. pyrifera* stipe and blade tissue collected from the surface canopy, mid canopy, and 1 m above the holdfast (*N* = 12 unique plants) to capture within‐plant variation in tissue dry mass composition (% wet mass). We used the slopes of the zero‐intercept linear regression lines generated from these relationships as conversion factors to calculate wet and dry mass for each surveyed plant from its frond density. Across all *M. pyrifera* tissue samples, wet biomass explained 96% of the variation in dry biomass. Although mean dry mass composition of *M. pyrifera* tissues varied by location along the frond, the range of total variation (8.8–12.6% of wet mass) was small. We chose to use a mean conversion value (10.3% of wet mass) to estimate dry mass for all *M. pyrifera* tissues, as we did not consistently collect the canopy length data necessary to incorporate within‐plant variation in dry mass composition. We summed the estimated dry mass of each plant and divided by surveyed area to calculate *M. pyrifera* FSC as dry mass (g · m^−2^) at each site for each survey.

We performed seasonal surveys of the understory stipitate kelp community, including *Neoagarum fimbriatum* and *Hedophyllum nigripes*, in July 2018–2020, January 2019–2020, and March 2019 at Breast Is., Harris Is., and Samsing Pinnacle. At each site, we counted individuals of these species within two permanent 30 × 2 m transects at the 5–7 m depth (MLLW) contour. Starting in March 2019, we also measured a subset of individuals for total blade length and maximum blade width. When we encountered >10 individuals of either species within a 10 × 1 m swath of a transect, we used the blade morphometrics calculated for the first 10 plants over a subsampled area to estimate total biomass for that species in the rest of that swath. To estimate total dry biomass from blade morphometrics, we collected >10 individuals of each understory kelp species from each site in August 2018, measured each blade for maximum length and width to estimate surface area (cm^−2^), and weighed for wet mass (g). We dried collected *Neoagarum fimbriatum* and *Hedophyllum nigripes* individuals at 60°C for at least 24 h and reweighed for dry mass (g). For each relationship (blade surface area to wet mass, and blade wet mass to dry mass), we used the slopes of the zero‐intercept linear regression lines as conversion factors to calculate a dry mass for each surveyed plant. Blade surface area explained 96% of the variability in thallus wet mass for *N. fimbriatum* and 97% of the variability in thallus wet mass for *H. nigripes* (Table S1). Thallus wet mass explained 99% of the variability in dry mass for both *N. fimbriatum* and *H. nigripes*.

We summed plant dry masses and divided by surveyed area to obtain the total dry mass FSC (g · m^−2^) of each understory species at each site for each survey. In instances where we performed surveys of both stipe counts and blade morphometrics during the same month, we used these calculated season‐specific relationships to estimate total dry mass FSC of each species from their stipe densities (stipes · m^−2^) prior to March 2019. We also used seasonal relationships between stipe counts or blade morphometrics and the season‐specific average wet mass of each understory kelp species to estimate the percent composition of understory FSC represented by each species in a survey. Stipe density in January 2020 explained 83% (*Neoagarum fimbriatum*) and 97% (*Hedophyllum nigripes*) of the variability in total dry mass present in the transect, whereas stipe counts in July 2019 and 2020 explained 53% (*N. fimbriatum*) and 98% (*H. nigripes*) of the variability in total thallus dry mass during these periods.

### Macroalgal growth and loss

We monitored monthly growth and loss of dominant kelp species in Sitka Sound from January 2017 to February 2018 at Breast Is. and Harris Is. (*Macrocystis pyrifera* only), and from July 2018 to July 2019 at Harris Is., Breast Is., and Samsing Pinnacle (*M. pyrifera*, *Neoagarum fimbriatum*, *Hedophyllum nigripes)*. At each site, we identified 12–15 “adult” individuals of each species (*M. pyrifera*: at least one frond reaching the surface; *N. fimbriatum* and *H. nigripes*: maximum blade length > 20 cm) along a 5–6 m depth (MLLW) contour with numbered tags. Each month, we re‐surveyed tagged *M. pyrifera* plants for frond density, with zip ties loosely bound around new fronds exceeding 1 m in height to distinguish new growth. For tagged *N. fimbriatum* and *H. nigripes* plants, each month we punched a new hole through the thallus at 10 cm from the intercalary meristem (Parke 1948), and we measured blade morphometrics (maximum blade length and width) and distance from meristem to the previous month's punched hole. When previously tagged individuals were not re‐sighted after two consecutive months, we assumed they had been physically removed from the substrate, either through grazing or abiotic factors.

We determined size‐specific growth and loss rates using an approach modified from Rassweiler et al. (2008, 2018). We use the term “size” broadly here, as we utilize either frond density (*Macrocystis pyrifera*) or blade length (understory species) to estimate sporophyte size as a proxy for sporophyte biomass. Because we use single conversion factor to calculate each species' sporophyte biomass from its size, size‐specific and mass‐specific growth rates are equivalent. Thus, hereafter we refer to them simply as “specific” rates. We calculated the specific frond loss or blade erosion rate (*f*_*i*_; d^−1^) of each plant during a survey period using the equation:

$$f_{i}=\frac{1}{T}\ln\left(\frac{F_{T}}{F_{0}}\right)$$

where *T* is the number of days between surveys, *F*_0_ is the frond density (*M. pyrifera*) or the maximum blade length (*Neoagarum fimbriatum*, *Hedophyllum nigripes)* at the start of the survey period (time 0), and *F*_*T*_ is the number of fronds >1 m that had zip ties at time 0 that remain at time *T* (*M. pyrifera*) or the maximum blade length at time 0 plus the difference between the total blade increase (maximum blade length at time *T* minus maximum blade length at time 0) and the linear blade growth (*N. fimbriatum*, *H. nigripes)*.

We calculated the specific growth rate (*g*_*i*_; d^−1^) of each plant during a survey period using the equation:

$$g_{i}=\frac{1}{T}\ln\left(\frac{B_{T}}{B_{0}}\right)+f_{i}$$

where *T* is the number of days between surveys, *B*_0_ is the frond density (*Macrocystis pyrifera*) or the maximum blade length (*Neoagarum fimbriatum*, *Hedophyllum nigripes*) at the start of the survey period (time *0*), and *B*_*T*_ is the total frond density or the maximum blade length at time *T*.

We calculated the per capita plant loss rate (*p*; d^−1^) for each species during a survey period using the equation:

$$p=\frac{1}{T}\ln\left(\frac{P_{T}}{P_{0}}\right)$$

where *T* is the number of days between surveys, *P*_0_ is the total number of individual plants of a species at the start of the survey period (time 0), and *P*_*T*_ is the number of plants at time 0 that remain at time *T*.

To determine a net rate of change (*n*; d^−1^) for all individuals of a species during a survey period, we calculated the difference between each individual's specific growth rate and the sum of the individual and species' loss rates*: n*_*i*_ = *g*_*i*_ − (*f*_*i*_ + *p*). We then averaged *n*_*i*_ among all individuals to get *n*. Similarly, we averaged *g*_*i*_ among individuals of each species during each survey period to calculate a mean specific growth rate (*g*).

Growth and loss equations were not defined in cases when all fronds were lost (*Macrocystis pyrifera*), or when the punched hole from time 0 was not re‐sighted at time *T* (*Neoagarum fimbriatum*, *Hedophyllum nigripes*). In the case of *M. pyrifera*, we substituted a value of ½ frond to enable an approximation of growth and loss rates as they approached zero (per Rassweiler et al. 2018). We did not observe any *M. pyrifera* plant to recover from a complete loss of fronds, and thus these individuals were accounted for in plant loss rates during a later survey period. When a punched hole was not re‐sighted on a tagged understory kelp species, we did not include the individual in our analyses for that survey period. Following our observations of multi‐year declines in *M. pyrifera* populations at two of our sites, we used regression analysis (R Core Team 2021) to test if the number of elapsed days in the study period was a significant predictor of *M. pyrifera* net growth rates at Harris and Breast Islands.

### Nutrient monitoring

To capture the annual variation in nutrient concentrations around a high latitude giant kelp bed, we sampled seawater monthly (July 2018 to July 2019) from the water column adjacent to Breast Is. in Sitka Sound, Alaska. We collected seawater using a surface‐deployed Niskin bottle at 0.5 m and 4.5 m depth at each of four locations: in the middle of the Breast Is. giant kelp bed canopy, at the canopy edge, 150 m away from canopy edge, and 600 m away from the canopy edge toward the open ocean (Gulf of Alaska). In addition, we collected benthic seawater samples monthly (June 2016 to July 2017) and opportunistically (fall 2017 to summer 2020) using a diver‐deployed Niskin bottle at 8–10 m depth at Breast Is., Harris Is., Samsing Pinnacle, and Talon Is. (57.073 N, 135.414 W). We brought collected water to the surface, immediately filtered each sample through a 0.2 μm filter and kept it frozen until analysis for dissolved inorganic nitrogen content as NO_*x*_ (NO_3_ + NO_2_) on a Lachat QuikChem 8000 Flow Injection Analyzer at the University of California Santa Cruz Marine Analytical Laboratory (detection limit <0.28 μM NO_*x*_, average run measurement error <0.1 μM NO_*x*_). To assess spatial variability in monthly seawater NO_*x*_ concentrations collected near Breast Is., we used a linear mixed‐effects model (R Core Team 2021) with depth, location, and the interaction of depth and location as fixed factors and date as a random intercept using restricted maximum likelihood. With log transformation of seawater NO_*x*_, we used plots of model residuals and Q–Q plots to confirm that our final model satisfied assumptions of homoskedasticity and normality (Winter 2013). We determined *P*‐values for the effects of fixed factors and their interactions using the Sattertwaithe's method for *t*‐tests (α = 0.05).

### Macroalgal carbon and nitrogen content

Coincident with monthly sampling of seawater for nutrient concentrations, we collected surface blades from *Macrocystis pyrifera* in the Breast Is. giant kelp bed from July 2018 to July 2019 to analyze for carbon (C) and nitrogen (N) content. On one frond from each plant (*N* = 3), we identified and removed the second intact blade closest to the frond's scimitar blade. To capture seasonal variation in C and N content in kelp species in July 2018, January 2019, and August 2019, we collected blades from *M. pyrifera* plants (*N* = 5) at ~1 m above their holdfasts and blades (*N* = 5) of *Neoagarum fimbriatum* and *Hedophyllum nigripes* between 4 and 7 m depth (MLLW) at Samsing Pinnacle. We also opportunistically collected blades (*N* = 3–5) representing all kelp species present at Harris Is. in summer 2018 and 2020 and at Samsing Pinnacle in summer 2020. For all macroalgal tissue field collections, we immediately drained collected samples of excess seawater and kept them on ice in a covered cooler for transport to the lab. Within 2 h of collection, we cleaned collected tissue of epiphytes and rinsed it briefly in fresh seawater. From all collected blades we excised 1–5 g of tissue immediately adjacent to the intercalary meristem where the blade meets the stipe. We spun tissue samples 10 times in a salad spinner before drying at 60°C for at least 24 h. Dried samples were analyzed for C and N content (% dry mass) by the University of California Santa Cruz Stable Isotope Laboratory using a CE Instruments NC2500 elemental analyzer coupled to a Thermo Scientific DELTAplus XP isotope ratio mass spectrometer via a Thermo‐Scientific Conflo III (routine measurement error ≤1.0%C and ≤0.2%N).

To assess the relationship between *Macrocystis pyrifera* surface blade N content and seawater NO_*x*_ concentration at Breast Is., we used a Spearman's rank correlation to compare blade tissue and seawater samples from 4.5 m depth (all seawater samples were pooled together by sampling date). We used two‐factor analysis of variance tests (R Core Team 2021) to analyze the effects of fixed factors *season* and *algal species* and the *interaction of season and species* on the C and N contents of *M. pyrifera*, *Hedophyllum nigripes*, and *Neoagarum fimbriatum* tissue collected at Samsing Pinnacle in 2018 and 2019. We confirmed assumptions of normality were met with *Q*–*Q* plots of model residuals, and used residual plots to verify the absence of heteroskedasticity (Winter 2013). Where fixed factors or their interaction were significant (ɑ = 0.05), we used the Tukey's honest significant difference method to test pairwise differences among means.

### Production estimates

We estimated macroalgal production rates in terms of dry mass, carbon mass, and nitrogen mass produced per square meter per day using a similar approach to Rassweiler et al. (2008, 2018). Calculations of giant kelp bed productivity in southern California were found to be robust to the type of growth model employed (Rassweiler et al. 2018). We chose to use an exponential growth model, which assumes that any new growth or erosion of a kelp sporophyte during a survey period occur in constant proportion to its starting size. For each survey period where we could estimate the starting dry mass FSC (*S*_0_; g · m^−2^) of a species at a site, we used the specific growth rate (*g*) and the specific net rate of change (*n*) to estimate the daily average dry mass production (*P*; g · m^−2^ · d^−1^) that occurred during this sampling interval:

$$P=\frac{g\cdot S_{0}}{n}\left(e^{n}-1\right)$$

We used the equation to calculate *P* in terms of carbon mass (i.e., net primary production or NPP) and nitrogen mass, except we first defined *S*_0_ in units of carbon or nitrogen mass by multiplying by the average carbon and nitrogen content of each species during that time period: *S*_0(C or N)_ = *S*_0_ · (%C or %N). We recognize the significant variation in C and N content that can exist within kelp thalli (Gevaert et al. 2001) and have confirmed inter‐thallus variability in elemental content for our monitored kelps in Sitka Sound that differs by species and season (L. Bell and K. Kroeker, unpub. data). Incorporation of this level of macroalgal elemental content variation into our productivity estimates was beyond the scope of this paper. We chose to use the average C and N content of the “newest” blade tissue (sampled closest to the intercalary meristem) as the sole conversion factor for each species in each time period. To calculate the error around our estimates of macroalgal production rates for each species at a site in a survey period, we used Monte Carlo methods to propagate uncertainty from measured variability in the actual data (Harmon et al. 2007). We generated 1000 randomly simulated normal distributions for each variable used in each calculation of *P* (as dry mass, C mass, and N mass) to create a normally distributed range of 1000 estimates of *P*. We then used the standard deviation of these values as the standard error in each of our estimates of *P*.

## RESULTS

### Plant biomass and foliar standing crop

**Fig. 1:**
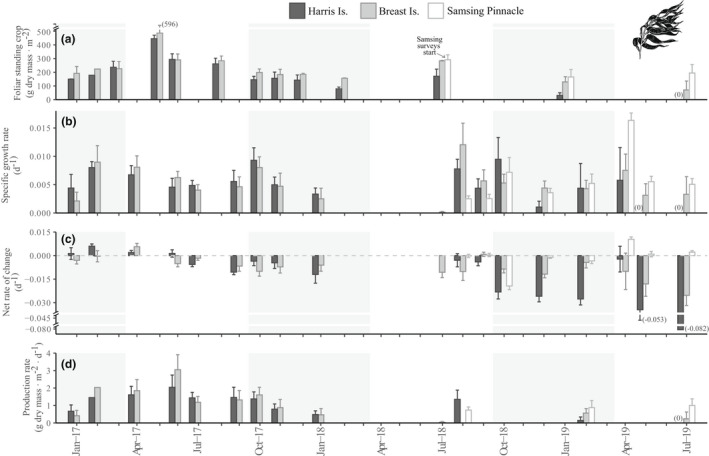
Site‐level estimates (mean ± SE) by survey period of *Macrocystis pyrifera* (a) foliar standing crop (g dry mass · m^−2^), (b) specific growth rate (d^−1^), (c) net rate of change (d^−1^), and (d) production rate (g dry mass · m^−2^ · d^−1^). A missing bar indicates no data for that particular site and survey period except where noted by “(0),” in which case the data point was zero. Shaded panels indicate the months with the shortest photoperiod (October–March).

From January 2017 to February 2018, *Macrocystis pyrifera* FSC was lowest in January (combined sites mean ± SE: 174 ± 24 g dry mass · m^−2^), but began to rise by April to reach an annual maximum around June (468 ± 47 g dry mass · m^−2^; Fig. 1a). By July, FSC had begun to decline again toward its winter minimum. At one site (Harris Is.), FSC was noticeably lower in July 2018 than had been observed during the same months in the prior year and continued to decline over the course of our study. By July 2019, giant kelp were absent along the surveyed transects at this site. A similar trend in declining *M. pyrifera* FSC was observed at a second site (Breast Is.) starting in slightly later (January 2019). Within 1 year of the noted decline (January 2020), there was a total loss of giant kelp from the surveyed area at this site (L. Bell and K. Kroeker, unpub. data). Concurrent with decreasing FSC, *M. pyrifera* mean plant density and average plant size (as number of fronds) also decreased at both sites.

**Fig. 2:**
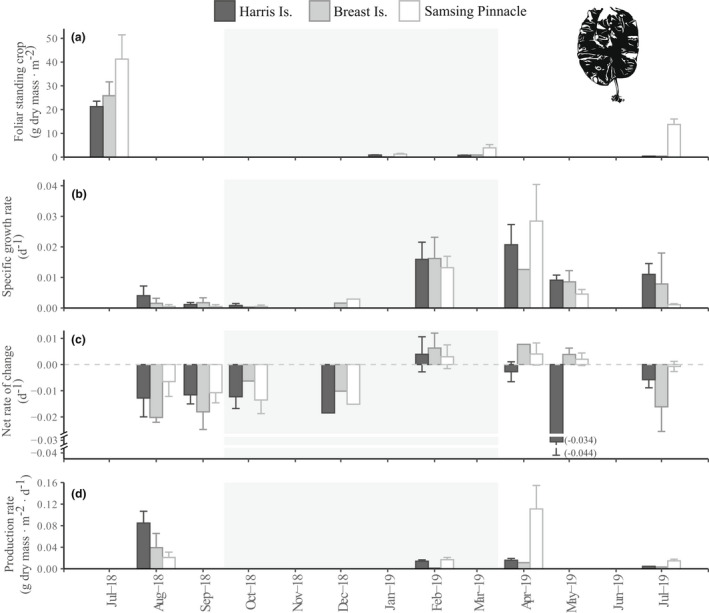
Site‐level estimates (mean ± SE) by survey period of *Hedophyllum nigripes* (a) foliar standing crop (g dry mass · m^−2^), (b) specific growth rate (d^−1^), (c) net rate of change (d^−1^), and (d) production rate (g dry mass · m^−2^ · d^−1^). A missing bar indicates no data for that particular site and survey period except where noted by “(0),” in which case the data point was zero. Shaded panel indicates the months with the shortest photoperiod (October–March).

**Fig. 3:**
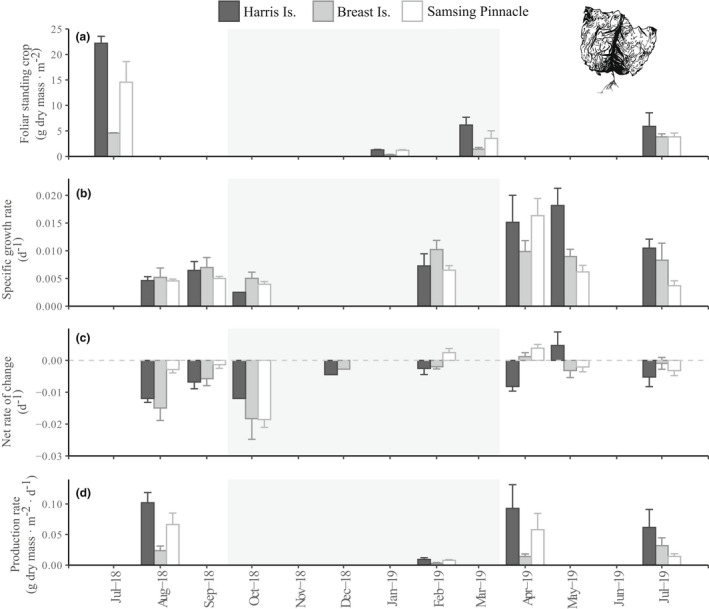
Site‐level estimates (mean ± SE) by survey period of *Neoagarum fimbriatum* (a) foliar standing crop (g dry mass · m^−2^), (b) specific growth rate (d^−1^), (c) net rate of change (d^−1^), and (d) production rate (g dry mass · m^−2^ · d^−1^). A missing bar indicates no data for that particular site and survey period except when noted by “(0),” in which case the data point was zero. Shaded panel indicates the months with the shortest photoperiod (October–March).

Estimated dry mass FSC of both *Hedophyllum nigripes* (Fig. 2a) and *Neoagarum fimbriatum* (Fig. 3a) were highest at all sites in July 2018. At Harris Is., *H. nigripes* and *N. fimbriatum* declined to local extinction over the course of our study (Table S1). Similarly, at Breast Is. *H. nigripes* was locally extinct from surveyed transects by January 2020 and *N. fimbriatum* had disappeared by January 2021 (L. Bell and K. Kroeker, unpub. data). Within the communities of understory kelps surveyed at each site, the species *Agarum clathratum* was consistently present in higher biomass than either *H. nigripes* and *N. fimbriatum*, but together these three species composed >97% of estimated total understory kelp FSC (as wet mass) during each survey at each site (Table S2 in the Supporting Information).

### Macroalgal growth and loss

At all sites, tagged *Macrocystis pyrifera* plants demonstrated new frond growth as well as frond loss in every surveyed period of this study (Fig. S1 in the Supporting Information). The one exception was at Harris Island in the final 2 months prior to site‐level extinction, where no new growth was observed on the few remaining *M. pyrifera* plants. Size‐specific growth rates of *M. pyrifera* appear to peak in the spring (during March and April; ~1.6% · d^−1^) and again in the early fall (~October; Fig. 1b). High frond and plant loss rates in the fall resulted in a mean negative net rate of change at all sites during October to December (Figs. 1c, S1). Net rates of change of *M. pyrifera* decreased over the duration of this study (February 2017 to August 2019) at both Harris Is. (simple linear regression model: *F*_1,15_ = 16.8, *P* < 0.001) and Breast Is. (simple linear regression model: *F*_1,16_ = 14.5, *P* = 0.002; Table S3 in the Supporting Information).

Both *Hedophyllum nigripes* and *Neoagarum fimbriatum* exhibited the highest specific growth rates in April–May (*H. nigripes*: ~2.8% · d^−1^; *N. fimbriatum*: ~1.6% · d^−1^) at all sites. The majority of *H. nigripes*' annual growth was observed in the first half of the calendar year (January to June; Fig. 2b). Compared with *H. nigripes*, tagged *N. fimbriatum* individuals sustained relatively higher specific growth rates through the late summer and fall (July to October; Fig. 3b). Both species experienced high erosion and plant loss rates in the late summer and fall (Figs. S2 and S3 in the Supporting Information), resulting in mean negative net rates of change at all sites during this period (Figs. 2c and 3c). We confirmed perennial recovery of both species from substantial grazing: tagged individuals that were observed in January with near‐complete blade loss and bearing characteristic grazing scars were re‐sighted in March with new growth of intact healthy blade tissue.

### Seawater nutrients

**Fig. 4:**
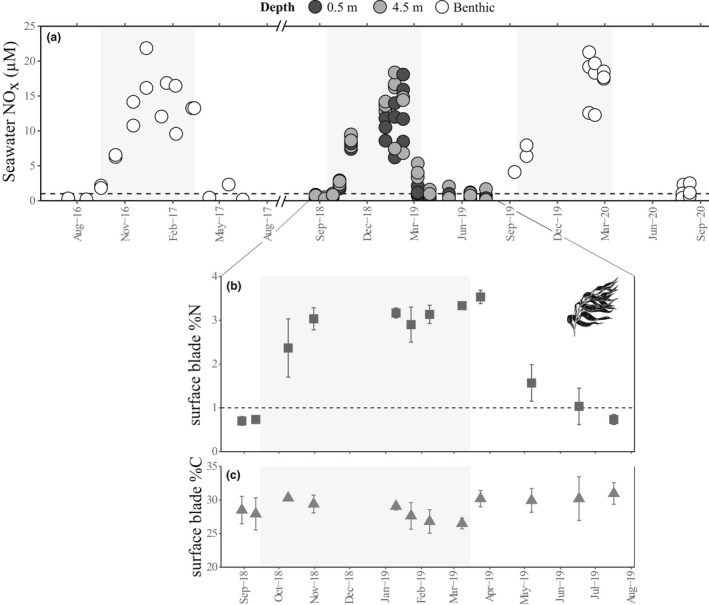
Annual variation in (a) seawater dissolved inorganic nitrogen as NO_*x*_ (μM) and in tissue (b) nitrogen and (c) carbon content of *Macrocystis pyrifera* surface canopy blades. Monthly from August 2018 to August 2019, kelp blades and water column seawater samples (0.5 m and 4.5 m depth) were collected on the same day at Breast Island. Outside of this time period, benthic seawater samples were collected opportunistically from kelp forest beds throughout Sitka Sound. Shaded panels indicate the months with the shortest photoperiod (October–March).

Seawater NO_*x*_ concentrations in Sitka Sound, Alaska followed a regular seasonal cycle, reaching their annual peak of 17–22 μM from December to February and remaining under 3 μM from April to August in each year of sampling (Fig. 4a). Water column NO_*x*_ concentrations sampled near the Breast Is. kelp bed were consistently higher at 4.5 m depth compared with 0.5 m (*P* < 0.001), but there was no relationship between nutrient concentration and location relative to the bed (mixed linear model: *F*_1,84_ = 0.67, *P* = 0.570) or the interaction between factors (mixed linear model: *F*_1,84_ = 1.49, *P* = 0.224; Table S4 in the Supporting Information).

### Macroalgal carbon and nitrogen content

The nitrogen (N) content of *Macrocystis pyrifera* surface canopy blades at Breast Is. in 2018–2019 were positively correlated with near‐surface seawater NO_*x*_ concentrations (Spearman's ρ = 0.72, *P* = 0.009; Fig. S4 in the Supporting Information). Outliers from samples collected in March, however, suggest that N content of *M. pyrifera* blades was decoupled from seawater nutrient concentrations in the spring for at least 1 month after NO_*x*_ began to decline (Fig. 4b). Surface blade N content reached its annual high in March (mean ± SE = 3.5 ± 0.1% dry mass) and an annual low in August (0.7 ± 0.1% dry mass). In contrast, *M. pyrifera* surface blade carbon (C) content remained relatively stable throughout the year at 29.0 ± 0.3% dry mass (Fig. 4c).

Nitrogen content of macroalgal tissue collected in 2018–2019 at Samsing Pinnacle was significantly impacted by the interaction between the effects of species and season (two‐way ANOVA: *F*_2,39_ = 15.5, *P* < 0.001; Tables S5 and S6 in the Supporting Information). Blade tissue N was higher in winter than summer for all three species (Tukey's HSD: *P* < 0.006). *Macrocystis pyrifera* had lower N content in the winter than either *Hedophyllum nigripes* (Tukey's HSD: *P* < 0.001) or *Neoagarum fimbriatum* (Tukey's HSD: *P* < 0.001), but the understory kelp species did not differ in N content from each other (Tukey's HSD: *P* = 0.406). In the summer, *N. fimbriatum* N content was higher than both *H. nigripes* (Tukey's HSD: *P* < 0.001) and *M. pyrifera* (Tukey's HSD: *P* < 0.001), whose N content did not differ from each other (Tukey's HSD: *P* = 0.851). Carbon content of kelp blade tissue collected during this period was impacted by season (two‐way ANOVA: *F*_1,39_ = 14.6, *P* < 0.001) and species (two‐way ANOVA: *F*_2,39_ = 11.2, *P* < 0.001), but we did not detect an interaction between these factors (two‐way ANOVA: *F*_2,39_ = 6.10, *P* = 0.005; Tables S5 and S7 in the Supporting Information). Tissue C did not differ between winter and summer seasons for *M. pyrifera* (Tukey's HSD: *P* = 0.582) or *N. fimbriatum* (Tukey's HSD: *P* = 0.999), but was higher in the summer compared with winter for *H. nigripes* (Tukey's HSD: *P* < 0.001). *Macrocystis pyrifera* had marginally lower C content in the winter than *N. fimbriatum* (Tukey's HSD: *P* = 0.044), as well as lower C content in the summer than *H. nigripes* (Tukey's HSD: *P* < 0.001), but otherwise within‐season blade C content did not differ among species (Tukey's HSD: *P* > 0.05).

### Production and turnover

Monthly monitoring of *Macrocystis pyrifera* beds in 2017–2018 indicated that annual dry mass productivity rates were maximal around June (mean ± SE: Harris Is.: 2.04 ± 0.70 g dry mass · m^−2^ · d^−1^; Breast Is.: 3.05 ± 0.86 g dry mass · m^−2^ · d^−1^) and minimum rates occurred around January (Fig. 1d). Giant kelp bed production rates at both Harris Is. and Breast Is. were comparatively lower in subsequent years and had dropped to zero at Harris Is. by July 2019. The highest productivity rate of *Hedophyllum nigripes* (0.11 ± 0.04 g dry mass · m^−2^ · d^−1^) was recorded in April 2019 at Samsing Pinnacle (Fig. 2d), whereas maximum productivity of *Neoagarum fimbriatum* (0.07 ± 0.02 g dry mass · m^−2^ · d^−1^) was observed in August 2018 at Samsing Pinnacle (Fig. 3d).

**Fig. 5:**
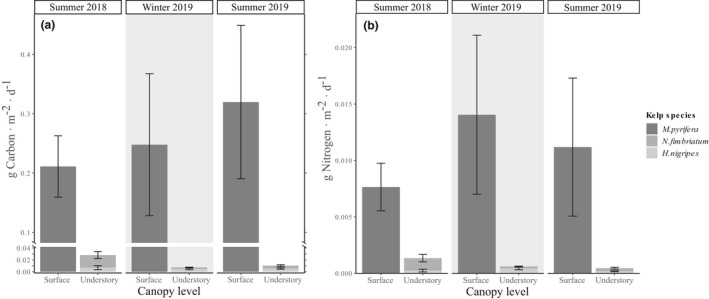
Seasonal production rates by canopy level for the giant kelp *Macrocystis pyrifera* and understory kelps *Hedophyllum nigripes* and *Neoagarum fimbriatum* by (a) carbon mass (g C · m^−2^ · d^−1^) and (b) nitrogen mass (g N · m^−2^ · d^−1^) at Samsing Pinnacle.

Estimated annual net primary production (C mass) in 2017 was ~142 g C · m^−2^ · year^−1^ at Harris Is. and ~ 156 g C · m^−2^ · year^−1^ at Breast Is. Using a ratio of total annual net primary production to the mean foliar standing crop at these sites in 2017 (Harris Is.: ~68 g C · m^−2^; Breast Is.: ~75 g C · m^−2^), we estimate the turnover of FSC in both of these *Macrocystis pyrifera* beds was approximately 2.1 times in that year. During seasonal sampling at Samsing Pinnacle in 2018–2019, mean C production rates of *M. pyrifera* ranged from 0.21 to 0.32 g C · m^−2^ · d^−1^, whereas estimated C production of *Hedophyllum nigripes* and *Neoagarum fimbriatum* combined did not exceed 0.03 g C · m^−2^ · d^−1^ in either season (Fig. 5a). In both winter and summer 2019, the total carbon mass production of the two understory kelp species represented less than 3.2% of *M. pyrifera* C production. The combined N mass production rates of *H. nigripes* and *N. fimbriatum* were 4.3% of estimated *M. pyrifera* N productivity in winter 2019, and 4.0% in summer 2019 (Fig. 5b). In summer 2018, there was a smaller relative difference in mass production rates between understory species and giant kelp, with C and N production by both *H. nigripes* and *N. fimbriatum* reaching 13.3% and 17.5%, respectively, of *M. pyrifera* C and N production.

## DISCUSSION

Here, we present the dry mass, carbon mass, and nitrogen mass production dynamics of three kelp species in a highly seasonal marine system. This work reveals the relative production rates of the surface canopy forming *Macrocystis pyrifera* and the two spatially dominant subcanopy kelps *Hedophyllum nigripes* and *Neoagarum fimbriatum* in giant kelp beds during periods of macroalgal persistence, as well as during phase shifts to urchin barrens. We calculate that the annual net primary production (g C · m^−2^ · year^−1^) of *M. pyrifera* in its polar fringe habitat is up to an order of magnitude lower than productivity estimates from the center of its range (Rassweiler et al. 2008, 2018, Reed et al. 2008, 2009). Foliar standing crop (FSC) and turnover rates are also nearly 2–3 times lower for *M. pyrifera* beds in Sitka Sound than mean values from southern California giant kelp forests. These results indicate that even ‘conservative’ estimates of *M. pyrifera* production in fringe habitats are currently too high and may therefore lead to overestimates of carbon flux through giant kelp forests in these regions (Reed and Brzezinski 2009, Wilmers et al. 2012, Duarte et al., 2022). Even so, production rates of giant kelp in this high latitude system dwarf total biomass contributions from co‐occurring understory kelp species *H. nigripes* and *N. fimbriatum*. These data are a valuable contribution to the limited year‐round studies of kelp growth and loss rates around the world and represent the highest latitude (N or S) consideration of *M. pyrifera* production rates yet (Pessarrodona et al. 2022).

Productivity of *Macrocystis pyrifera* in its fringe habitat compared with range center populations is likely primarily constrained by light availability (Stekoll and Else 1990, Graham et al. 2007, Stekoll et al. 2021), although seasonal nutrient limitation may also play a role (van Tussenbroek 1989). Our monthly seawater nutrient monitoring confirms that subtidal macroalgae in this system have access to high NO_*x*_ concentrations (>5 μM) from October through March. However, nutrient depletion from enhanced water column production in the spring brings these concentrations below 1 μM, the putative minimum concentration necessary to sustain *M. pyrifera* growth (Gerard 1982a). Our sampling of *M. pyrifera* surface blades indicates a 2‐month lag between the decline in seawater NO_*x*_ and a decrease in the nitrogen content of their tissues. Unlike giant kelp in southern California that maintain reserves of nitrogen in their tissues throughout the year (Stewart et al. 2009, Brzezinksi et al. 2013), *M. pyrifera* in Sitka Sound experience a 2‐month period in the late summer during which their blade nitrogen reserves are depleted (blade % N below 1% dry mass; Gerard 1982b). This seasonal nutrient deficiency corresponds with seasonal lows in specific growth and productivity rates of *M. pyrifera* in Sitka Sound, similar to giant kelp ecosystems in coastal waters of New Zealand and the Falkland Islands (van Tussenbroek 1989, Brown et al. 1997).

Monthly tagging of *Hedophyllum nigripes* and *Neoagarum fimbriatum* reveals that the annual growth cycles for these understory species also follow seasonal variation in light and nutrient availability in this system. Although external NO_*x*_ sources have declined by March, these understory kelps are likely able to draw on internal nitrogen reserves accumulated during winter for up to 1–2 months before this source is depleted (Korb and Gerard 2000, Pueschel and Korb 2001). Relative nitrogen storage capacity and rate of utilization may underlie slight differences in annual growth regime between the two species. In January at Samsing Pinnacle, both *H. nigripes* and *N. fimbriatum* had similarly high tissue nitrogen content ahead of notable spring increases in their growth and production rates. However, by summer, *N. fimbriatum* individuals maintained both higher relative tissue nitrogen content than *H. nigripes* as well as higher relative growth from July to October. Further, decreased carbon content in *H. nigripes* in winter relative to summer may indicate that this species utilizes a substantial proportion of its carbohydrate reserves for growth as early as January, at a time that loss of carbon through respiration exceeds assimilation of new carbon via photosynthesis (Gómez and Wiencke 1998, Gevaert et al. 2001). Such species‐specific differences suggest that these co‐occurring understory kelps employ distinct ‘strategies’ in the timing and magnitude of their resource mobilization for growth. In this system, these phenological differences in basal production may be critical to sustaining certain consumers' energetic demands during the more physiologically stressful winter season (Kroeker et al. 2021).

Variability in relative carbon and nitrogen production rates among kelp species suggests that the importance of understory kelps as a potential food source to marine consumers may also vary markedly inter‐annually. Regardless of season, *Macrocystis pyrifera* dominated total C and N production by kelp at Samsing Pinnacle. However, both understory species exhibit equal or higher relative C and N concentrations per tissue mass than *M. pyrifera*. As a result, when understory kelp FSC was relatively high (such as during summer 2018), the relative proportion of C and N production by these species compared to giant kelp was notably increased. Prior work in this system has shown that pinto abalone (*Haliotis kamtschatkana*), a common rocky reef grazer, experience higher growth rates when fed a mixed algal diet consisting of several kelp species than when fed a diet of *M. pyrifera* alone (Kroeker et al. 2021). Given that *M. pyrifera* dwarfed understory kelp species in terms of both FSC and productivity at sites not undergoing phase shifts, even modest increases in the relative productivity of any understory kelp species would provide a valuable, diverse source of nutrition to the primary consumer community.

Our consideration of only two understory kelp species means that our results certainly underestimate total dry mass production by understory kelps in this system. Based on our community surveys, *Hedophyllum nigripes* and *Neoagarum fimbriatum* only composed 30% of total understory kelp wet mass FSC in some seasons. Inclusion of the biomass‐dominant, C‐ and N‐rich understory kelp *Agarum clathratum* in our study would have provided a more complete picture of understory kelp production dynamics. We have observed that *A. clathratum* is not readily consumed by grazers, perhaps due high polyphenolic concentrations or tissue toughness affecting its palatability relative to other kelps (Alstyne et al. 1999, Dubois and Iken 2012). It is consistently the last kelp species to be grazed on rocky reefs undergoing phase shifts from kelp beds to urchin barrens in Sitka Sound (L. Bell, pers. obs.). Therefore, although C and N production represented by *H. nigripes* and *N. fimbriatum* accounts for the bulk of the understory kelp production important to rocky reef grazers, future work considering the production of *A. clathratum* will be essential to predicting the potential C cycling and sequestration capacity of understory kelp communities in this region.

Our estimates of frond, blade, and whole‐plant loss rates highlight the year‐round turnover of macroalgal biomass in this system. In addition, we confirm that the late fall and early winter seasons represent a regular period of enhanced net tissue loss for all three kelp species. Winter storm swell in the North Pacific is high during this time and can drive whole plant losses via mechanical stress on holdfasts (Druehl and Wheeler 1986, Pedersen et al. 2020). We also observed substantial blade loss via grazing during the late fall and early winter. Unfortunately, we are unable to tease apart how much of the net tissue loss at this time of year is due to increased grazing pressure versus decreased algal growth. On tagged understory kelps, such intense grazing frequently extended past the prior survey's punched hole, making it challenging to quantify blade growth and loss rates during these seasons. Even so, our calculations of high loss rates of detrital and particulate matter appear to be on par with kelp from other high latitude regions, representing substantial carbon supply to the surrounding marine ecosystem (Krause‐Jensen and Duarte 2016, Pedersen et al. 2020, Smale et al. 2021). And our study did not account for losses of macroalgal tissue released as dissolved organic carbon, which can represent an estimated 13–35% of fixed carbon in kelps (reviewed by Paine et al. 2021). Because we did not track the fate of all ‘lost’ macroalgal production, we cannot accurately assess the carbon sequestration potential of these kelp species (Hurd et al. 2022).

We acknowledge that our seaweed survey methods could not capture all new tissue growth, which would explain how these populations could persist despite our calculations of negative net rates of change during the majority of the year. For tagged *Macrocystis pyrifera* individuals, our periodic surveys would have missed any new fronds that grew and were subsequently lost in between sampling periods. For *Neoagarum fimbriatum* and *Hedophyllum nigripes*, our hole‐punch method could not account for any growth that occurred beyond the punched hole, which is known to occur in stipitate kelps (Calvin and Ellis 1981, Gagné and Mann 1987, Miller et al. 2011). Furthermore, kelps can invest new growth in increasing blade width and thickness (Calvin and Ellis 1981, Druehl et al. 1987) as well as stipe mass (Gagné and Mann 1987), and none of these metrics were captured in our tagged understory surveys. Lastly, we did not incorporate any temporal or spatial variation in the conversion factors used to calculate each species' tissue dry mass from wet mass and blade surface area measurements. Our assumption of low variability in these relationships is supported by prior studies in certain kelp species (Rassweiler et al. 2018, Wickham et al. 2019) but not others (Gagné et al. 1982, Gevaert et al. 2001). For that reason we advise that our coarse estimates of species' net rates of change at each site be interpreted alongside a consideration of the variation in species' FSC over time.

At two of our monitored sites, FSC of *Macrocystis pyrifera* and *Hedophyllum nigripes* did unexpectedly decline over the course of our study. By 2021, all kelps except for *Agarum clathratum* had disappeared from these sites. Previous studies in this area suggest that these site‐level phase shifts occurred due to changes in top‐down pressures (Raymond et al. 2019, Gorra et al. 2022). Recent marine heatwaves and sea star wasting in this region may have also influenced the structure of these rocky reef communities (Burt et al. 2018, Ross et al. 2021). However, environmental and invertebrate community monitoring data collected at these sites do not indicate notable differences in annual temperatures or sea star abundances that correlate with patterns of macroalgal loss (L. Bell and K. Kroeker, unpub. data). The spatial pattern of kelp forest declines that we have recently observed in Sitka Sound (e.g., kelp forests transitioning to barrens predominantly in areas of high human activity) suggests that human‐influenced trophic cascades were a primary driver of change at our sites. At Harris Is., where we first noticed the net rate of change of *M. pyrifera* (and later, *H. nigripes*) becoming unusually negative, there appeared to be a simultaneous increase in the mean and variability of these species' specific growth rates. This short‐term pattern may have arisen from decreased competition for resources (e.g., light and nutrients) as FSC of canopy‐forming conspecifics declined (Gerard 1976, Reed et al. 2008). Although increased resource availability following removal of a *M. pyrifera* surface canopy can enhance understory kelp production in some cases (Miller et al. 2011, Castorani et al. 2021), we did not observe such a response at our sites. This finding is consistent with research in southern California showing that high herbivore densities can suppress the response of understory algae to surface canopy loss (Castorani et al. 2021). Net loss rates of each kelp species eventually overwhelmed any temporary increases in their specific growth rates, and the majority of the kelp carbon mass lost during these phase shifts was likely consumed and remineralized as CO_2_ (Krause‐Jensen and Duarte 2016, Filbee‐Dexter and Wernberg 2020). The complete eradication of the *M. pyrifera* population at both Harris Is. and Breast Is. represents approximately 150 g C · m^−2^ · year^−1^ of lost production from giant kelp alone. While the loss of canopy‐forming macroalgal species can benefit local phytoplankton productivity, phytoplankton are unable to fully compensate for the production capacity of these biomass‐rich kelp beds (Pfister et al. 2019). Therefore, the loss of these macroalgal communities represents a net decrease in the carbon sequestration capacity of these coastal rocky reef areas (Wilmers et al. 2012, Gorra et al. 2022).

Current debate over the relevance of macroalgae to global blue carbon stocks has resulted in a demand for more robust accounting of carbon flows through seaweed beds (Macreadie et al. 2019, Bach et al. 2021, Gallagher et al. 2022, Hurd et al. 2022). Our results underscore the importance of integrating productivity estimates for each species from a diversity of environments in order to accurately assess its aggregate potential contribution to carbon and nitrogen storage and cycling. We calculate that the production rates of the globally distributed foundational kelp *Macrocystis pyrifera* are substantially lower at the poleward fringe of its range compared with populations from its range center. We also provide the first estimates of production capacity for the subcanopy kelps *Hedophyllum nigripes* and *Neoagarum fimbriatum* associated with high latitude giant kelp beds, which represent only 3–18% of *M. pyrifera* production in winter or summer. These findings indicate that the kelps composing high latitude *M. pyrifera* beds may not contribute substantially to global kelp production, as their productivity falls substantially below even the lower‐bound estimates for this ecosystem (Reed and Brzezinski 2009).

Our consideration of kelp production capacity in high latitude *Macrocystis pyrifera* beds comes at a time of dramatic change in these marine environments. Polar regions are experiencing some of the fastest rates of ocean warming and acidification in the world (Fabry et al. 2009, Mathis et al. 2015, IPCC 2018). The global geographic distribution of kelp communities is shifting, with some of the most dramatic changes to kelp abundance projected at species' poleward edges (Krumhansl et al. 2016, Smale 2020). Concurrently, there is heightened interest in seaweed mariculture and macroalgal carbon sequestration potential in these high latitude regions (AMTF 2018, Stekoll 2019, Smale et al. 2021). Understanding the relative timing and magnitude of production among kelp species in naturally occurring beds is an essential first step to predicting how future global change could affect these significant basal energy sources. Although not considered in our study, the bull kelp *Nereocystis luetkeana* may currently have higher carbon fixation and dissolved carbon release than *M. pyrifera* where they co‐occur in the north Pacific (Weigel and Pfister 2021). However, if the warm‐temperate adapted *M. pyrifera* increases in abundance due to favorable environmental changes, it may be better poised to outcompete and outperform cold‐temperate adapted kelp assemblages in production capacity (as has been seen in climate‐driven kelp community changes in the NE Atlantic; Pessarrodona et al. 2019). Additionally, future increases in ocean temperatures and *p*CO_2_ have the potential to alter the assimilation and elemental composition of these macroalgae as well as their rates of organic matter release (Pessarrodona et al. 2018, Close et al. 2020, Paine et al. 2021, Wright et al. 2022). Research investigating how such changes in the marine environment will impact the carbon and nitrogen mass productivity of these coastal primary producers will be a crucial next step for predicting the future carbon sequestration potential of high latitude kelp forest communities (Harley et al. 2012, Gilson et al. 2021).

## Supporting information


**Figure S1.** Per‐plant proportions of *Macrocystis pyrifera* fronds grown or fronds lost compared with starting frond density (mean ± SE; top panel) and the site‐level plant loss rate (bottom panel) during each survey period at (a) Breast Is., (b) Harris Is. and (c) Samsing Pinnacle. A missing bar indicates no data for that particular site and survey period except where noted by “(0),” in which case the data point was zero. Shaded panels indicate the months with the shortest photoperiod (October–March).

**Figure S2.** Linear growth and erosion rates (cm · d^−1^) of *Hedophyllum nigripes* blades (mean ± SE; top panel) and the site‐level plant loss rate (bottom panel) during each survey period at (a) Breast Is., (b) Harris Is. and (c) Samsing Pinnacle. A missing bar indicates no data for that particular site and survey period except where noted by “(0),” in which case the data point was zero. Shaded panel indicates the months with the shortest photoperiod (October–March).

**Figure S3.** Linear growth and erosion rates (cm · d^−1^) of *Neoagarum fimbriatum* blades (mean ± SE; top panel) and the site‐level plant loss rate (bottom panel) during each survey period at (a) Breast Is., (b) Harris Is. and (c) Samsing Pinnacle. A missing bar indicates no data for that particular site and survey period except where noted by “(0),” in which case the data point was zero. Shaded panel indicates the months with the shortest photoperiod (October–March).

**Figure S4.** Spearman rank correlation scatter plot for log‐transformed seawater NO_*x*_ concentrations (μM) from 4.5 m depth versus nitrogen content (as % dry mass) of *M. pyrifera* surface blades at Breast Is (mean ± SE). Linear regression and 95% confidence interval are shown as the gray line and shaded region. Spearman's rank correlation (ρ) and associated p‐value are shown in upper left corner.

**Table S1.** Regression parameters used to estimate macroalgal wet and dry mass for foliar standing crop determination.

**Table S2.** Estimated foliar standing crop (g wet mass · m^−2^) of subtidal understory kelp species by survey site and season from blade morphometric surveys.

**Table S3.** Regression parameters used to test the effect of elapsed days in the study on *Macrocystis pyrifera* growth rate at two sites.

**Table S4.** Summary statistics from mixed linear model analysis of monthly seawater NO_*x*_ concentrations near Breast Is. Formula: log(seawater NO_*x*_) ~ depth * location + (1¦date).

**Table S5.** Elemental composition (carbon or nitrogen as % dry mass) of subtidal kelp species by collection site and season.

**Table S6.** Summary statistics for analysis of variance of macroalgal tissue nitrogen concentrations at Samsing Pinnacle Formula: nitrogen (as % dry mass) ~ season * species.

**Table S7.** Summary statistics for analysis of variance of macroalgal tissue carbon concentrations at Samsing Pinnacle Formula: carbon (as % dry mass) ~ season * species.

## Data Availability

The data that support the findings of this study are openly available in BCO‐DMO at www.bco‐dmo.org, project number 756735.
