# Supplementary material for: Standing Crop, Turnover, and Production Dynamics of Macrocystis pyrifera and Understory Species Hedophyllum nigripes and Neoagarum fimbriatum in High Latitude Giant Kelp Forests
Source: J Phycol. 2022 Nov 17;58(6):773–88. doi: 10.1111/jpy.13291 (PMC10100489; doi:10.1111/jpy.13291)
Supplement: Supplementary file 1 — Figure S1. Per‐plant proportions of Macrocystis pyrifera fronds grown or fronds lost compared with starting frond density (mean ± SE; top panel) and the site‐level plant loss rate (bottom panel) during each survey period at (a) Breast Is., (b) Harris Is. and (c) Samsing Pinnacle. A missing bar indicates no data for that particular site and survey period except where noted by “(0),” in which case the data point was zero. Shaded panels indicate the months with the shortest photoperiod (October–March). [file JPY-58-773-s006.docx]

Figure S1. Per-plant proportions of *Macrocystis pyrifera* fronds grown or fronds lost compared to starting frond density (mean ± SE; top panel) and the site-level plant loss rate (bottom panel) during each survey period at (a) Breast Is., (b) Harris Is. and (c) Samsing Pinnacle. A missing bar indicates no data for that particular site and survey period except where noted by “(0)”, in which case the data point was zero. Shaded panels indicate the months with the shortest photoperiod (October – March).
