# Supplementary material for: Standing Crop, Turnover, and Production Dynamics of Macrocystis pyrifera and Understory Species Hedophyllum nigripes and Neoagarum fimbriatum in High Latitude Giant Kelp Forests
Source: J Phycol. 2022 Nov 17;58(6):773–88. doi: 10.1111/jpy.13291 (PMC10100489; doi:10.1111/jpy.13291)
Supplement: Supplementary file 3 — Figure S3. Linear growth and erosion rates (cm · d−1) of Neoagarum fimbriatum blades (mean ± SE; top panel) and the site‐level plant loss rate (bottom panel) during each survey period at (a) Breast Is., (b) Harris Is. and (c) Samsing Pinnacle. A missing bar indicates no data for that particular site and survey period except where noted by “(0),” in which case the data point was zero. Shaded panel indicates the months with the shortest photoperiod (October–March). [file JPY-58-773-s011.docx]

Figure S3. Linear growth and erosion rates (cm · d^-1^) of *Neoagarum fimbriatum* blades (mean ± SE; top panel) and the site-level plant loss rate (bottom panel) during each survey period at (a) Breast Is., (b) Harris Is. and (c) Samsing Pinnacle. A missing bar indicates no data for that particular site and survey period except where noted by “(0)”, in which case the data point was zero. Shaded panel indicates the months with the shortest photoperiod (October – March).
