# Supplementary material for: Standing Crop, Turnover, and Production Dynamics of Macrocystis pyrifera and Understory Species Hedophyllum nigripes and Neoagarum fimbriatum in High Latitude Giant Kelp Forests
Source: J Phycol. 2022 Nov 17;58(6):773–88. doi: 10.1111/jpy.13291 (PMC10100489; doi:10.1111/jpy.13291)

Figure S4. Spearman rank correlation scatter plot for log-transformed seawater NOx concentrations (µM) from 4.5 m depth versus nitrogen content (as % dry mass) of *M. pyrifera* surface blades at Breast Is (mean ± SE). Linear regression and 95% confidence interval are shown as the gray line and shaded region. Spearman’s rank correlation (ρ) and associated p-value are shown in upper left corner.


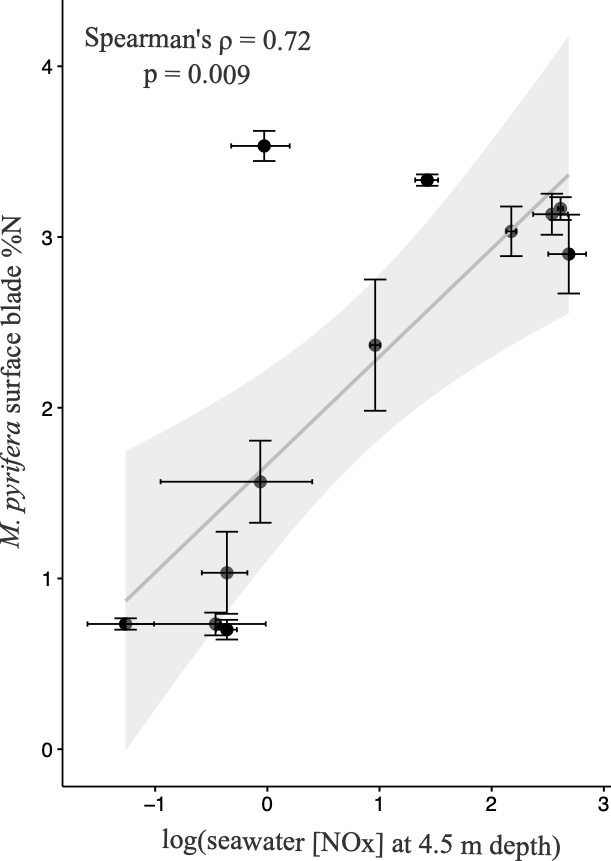

Supplement: Supplementary file 4 — Figure S4. Spearman rank correlation scatter plot for log‐transformed seawater NO x concentrations (μM) from 4.5 m depth versus nitrogen content (as % dry mass) of M. pyrifera surface blades at Breast Is (mean ± SE). Linear regression and 95% confidence interval are shown as the gray line and shaded region. Spearman's rank correlation (ρ) and associated p‐value are shown in upper left corner. [file JPY-58-773-s002.docx]
