# Supplementary material for: Standing Crop, Turnover, and Production Dynamics of Macrocystis pyrifera and Understory Species Hedophyllum nigripes and Neoagarum fimbriatum in High Latitude Giant Kelp Forests
Source: J Phycol. 2022 Nov 17;58(6):773–88. doi: 10.1111/jpy.13291 (PMC10100489; doi:10.1111/jpy.13291)
Supplement: Supplementary file 5 — Table S1. Regression parameters used to estimate macroalgal wet and dry mass for foliar standing crop determination. [file JPY-58-773-s001.docx]

Table S1. Regression parameters used to estimate macroalgal wet and dry mass for foliar standing crop determination

| **Independent_variable** | **Dependent_variable** | **slope** | **intercept** | **pvalue** | **rsquare** | **N** | **Sample unit** |
| --- | --- | --- | --- | --- | --- | --- | --- |
| *M. pyrifera* frond density (all seasons) | *M. pyrifera* wet mass (g; all seasons) | 734.9 | 0 | <0.001 | 0.943 | 26 | individual sporophytes |
| *M. pyrifera* frond density (winter only) | *M. pyrifera* wet mass (g; winter only) | 748.2 | 0 | <0.001 | 0.926 | 10 | individual sporophytes |
| *M. pyrifera* frond density (summer only) | *M. pyrifera* wet mass (g; summer only) | 732.9 | 0 | <0.001 | 0.941 | 16 | individual sporophytes |
| *M. pyrifera* wet mass (g; all tissue samples) | *M. pyrifera* dry mass (g; all tissue samples) | 0.103 | 0 | <0.001 | 0.962 | 68 | tissue samples |
| *M.pyrifera* wet mass (g; stipe tissue only) | *M. pyrifera* dry mass (g; stipe tissue only) | 0.110 | 0 | <0.001 | 0.963 | 36 | tissue samples |
| *M.pyrifera* wet mass (g; blade tissue only) | *M. pyrifera* dry mass (g; blade tissue only) | 0.096 | 0 | <0.001 | 0.968 | 32 | tissue samples |
| *M.pyrifera* wet mass (g; surface tissue only) | *M. pyrifera* dry mass (g; surface tissue only) | 0.088 | 0 | <0.001 | 0.974 | 23 | tissue samples |
| *M.pyrifera* wet mass (g; mid-frond tissue only) | *M. pyrifera* dry mass (g; mid-frond tissue only) | 0.095 | 0 | <0.001 | 0.981 | 24 | tissue samples |
| *M.pyrifera* wet mass (g; tissue 1m from HF only) | *M. pyrifera* dry mass (g; tissue 1m from HF only) | 0.126 | 0 | <0.001 | 0.988 | 21 | tissue samples |
| *N. fimbriatum* blade surface area (cm^2^) | *N. fimbriatum* blade wet mass (g) | 0.066 | 0 | <0.001 | 0.962 | 40 | individual sporophytes |
| *H. nigripes* blade surface area (cm^2^) | *H. nigripes* blade wet mass (g) | 0.092 | 0 | <0.001 | 0.970 | 41 | individual sporophytes |
| *A. clathratum* blade surface area (cm^2^) | *A. clathratum* blade wet mass (g) | 0.131 | 0 | <0.001 | 0.925 | 20 | individual sporophytes |
| *L. setchellii* blade surface area (cm^2^) | *L. setchellii* blade wet mass (g) | 0.097 | 0 | <0.001 | 0.983 | 5 | individual sporophytes |
| *P. gardneri* blade surface area (cm^2^) | *P. gardneri* blade wet mass (g) | 0.080 | 0 | 0.066 | 0.801 | 3 | individual sporophytes |
| *N. fimbriatum* blade wet mass (g) | *N. fimbriatum* blade dry mass (g) | 0.136 | 0 | <0.001 | 0.994 | 10 | individual sporophytes |
| *H. nigripes* blade wet mass (g) | *H. nigripes* blade dry mass (g) | 0.190 | 0 | <0.001 | 0.987 | 11 | individual sporophytes |
| *N. fimbriatum* stipe density (# m^-2^), Jan 2020 | *N. fimbriatum* dry biomass (g m-2), Jan 2020 | 2.505 | 0 | 0.007 | 0.825 | 5 | transects |
| *H. nigripes* stipe density (# m^-2^), Jan 2020 | *H. nigripes* dry biomass (g m-2), Jan 2020 | 5.883 | 0 | 0.001 | 0.971 | 4 | transects |
| *N. fimbriatum* stipe density (# m^-2^), July 2019 & 2020 | *N. fimbriatum* dry biomass (g m-2), July 2019 & 2020 | 17.691 | 0 | 0.016 | 0.525 | 8 | transects |
| *H. nigripes* stipe density (# m^-2^), July 2019 & 2020 | *H. nigripes* dry biomass (g m-2), July 2019 & 2020 | 49.994 | 0 | <0.001 | 0.982 | 8 | transects |
