# Supplementary material for: Standing Crop, Turnover, and Production Dynamics of Macrocystis pyrifera and Understory Species Hedophyllum nigripes and Neoagarum fimbriatum in High Latitude Giant Kelp Forests
Source: J Phycol. 2022 Nov 17;58(6):773–88. doi: 10.1111/jpy.13291 (PMC10100489; doi:10.1111/jpy.13291)
Supplement: Supplementary file 6 — Table S2. Estimated foliar standing crop (g wet mass · m−2) of subtidal understory kelp species by survey site and season from blade morphometric surveys. [file JPY-58-773-s009.docx]

Table S2. Estimated foliar standing crop (g wet mass · m^-2^) of subtidal understory kelp species by survey site and season from blade morphometric surveys.

|  | **Breast** | | | | **Harris** | | | | **Samsing** | | | |
| --- | --- | --- | --- | --- | --- | --- | --- | --- | --- | --- | --- | --- |
|  | 2019 | | 2020 | | 2019 | | 2020 | | 2019 | | 2020 | |
| **Species** | Spring | Summer | Winter | Summer | Spring | Summer | Winter | Summer | Spring | Summer | Winter | Summer |
| *Hedophyllum nigripes* | 1.84 | 0^a^ | 0 | 0 | 2.48 | 0.49 | 0 | 0 | 17.78 | 114.74 | 13.59 | 86.21 |
| *Neoagarum fimbriatum* | 5.12 | 22.17 | 0.36 | 0.47 | 19.87 | 34.97 | 0.12 | 0 | 12.84 | 60.66 | 3.89 | 38.70 |
| *Agarum clathratum* | 0 | 0 | 0 | 0 | 57.40 | 82.55 | 11.34 | 21.68 | 33.47 | 171.51 | 24.18 | 107.26 |
| *Laminaria setchellii* | 0 | 0 | 0 | 0 | 0 | 0 | 0 | 0 | 0.88 | 3.17 | 0.02 | 3.44 |
| *Pleurophycus gardeneri* | 0 | 0 | 0 | 0 | 0 | 0 | 0 | 0 | 0.18 | 3.33 | 0 | 5.11 |
| **Total understory kelp biomass (g· m^-2^)** | **6.96** | **22.17** | **0.36** | **0.47** | **79.75** | **118.01** | **11.46** | **21.68** | **65.15** | **353.41** | **41.67** | **240.73** |

^a^Some plants tagged for productivity measurements did still exist at this site in this season, but were not captured in this particular survey
