# Supplementary material for: Standing Crop, Turnover, and Production Dynamics of Macrocystis pyrifera and Understory Species Hedophyllum nigripes and Neoagarum fimbriatum in High Latitude Giant Kelp Forests
Source: J Phycol. 2022 Nov 17;58(6):773–88. doi: 10.1111/jpy.13291 (PMC10100489; doi:10.1111/jpy.13291)
Supplement: Supplementary file 7 — Table S3. Regression parameters used to test the effect of elapsed days in the study on Macrocystis pyrifera growth rate at two sites. [file JPY-58-773-s005.docx]

Table S3. Regression parameters used to test the effect of elapsed days in the study on *M. pyrifera* growth rate at two sites

| **Independent_variable** | **Dependent_variable** | **slope** | **intercept** | **pvalue** | **rsquare** | **N** | **Sample unit** |
| --- | --- | --- | --- | --- | --- | --- | --- |
| Number of elapsed days in the study | *M. pyrifera* net growth rate (d^-1^; Harris Is.) | -0.00003 | 0.005 | <0.001 | 0.497 | 16 | site surveys |
| Number of elapsed days in the study | *M. pyrifera* net growth rate (d^-1^; Breast Is.) | -0.00001 | 0.00008 | 0.002 | 0.442 | 17 | site surveys |
