# Supplementary material for: Standing Crop, Turnover, and Production Dynamics of Macrocystis pyrifera and Understory Species Hedophyllum nigripes and Neoagarum fimbriatum in High Latitude Giant Kelp Forests
Source: J Phycol. 2022 Nov 17;58(6):773–88. doi: 10.1111/jpy.13291 (PMC10100489; doi:10.1111/jpy.13291)
Supplement: Supplementary file 8 — Table S4. Summary statistics from mixed linear model analysis of monthly seawater NO x concentrations near Breast Is. Formula: log(seawater NO x ) ~ depth * location + (1¦date). [file JPY-58-773-s010.docx]

Table S4. Summary statistics from mixed linear model analysis of monthly seawater NO_x_ concentrations near Breast Is.

Formula: log(seawater NO_x_) ~ depth * location + (1|date)

i. *Variance components for random effects*

| **Groups** | **Variance** | **Std. Dev.** |
| --- | --- | --- |
| date | 2.244 | 1.498 |
| residual | 0.237 | 0.487 |

*Number of observations: 104*

*Groups: date, 13*

ii*. ANOVA results from the mixed linear model*

| **Source** | **SS** | **MSE** | **numDF** | **denDF** | **F value** | **Pr(>F)** |
| --- | --- | --- | --- | --- | --- | --- |
| depth | 3.030 | 3.030 | 1 | 84.020 | 12.771 | <0.001 |
| location | 0.480 | 0.160 | 3 | 84.027 | 0.674 | 0.570 |
| depth:location | 1.057 | 0.352 | 3 | 84.018 | 1.486 | 0.224 |
