# Supplementary material for: Standing Crop, Turnover, and Production Dynamics of Macrocystis pyrifera and Understory Species Hedophyllum nigripes and Neoagarum fimbriatum in High Latitude Giant Kelp Forests
Source: J Phycol. 2022 Nov 17;58(6):773–88. doi: 10.1111/jpy.13291 (PMC10100489; doi:10.1111/jpy.13291)
Supplement: Supplementary file 9 — Table S5. Elemental composition (carbon or nitrogen as % dry mass) of subtidal kelp species by collection site and season. [file JPY-58-773-s004.docx]

Table S5. Elemental composition (carbon or nitrogen as % dry mass) of subtidal kelp species by collection site and season.

| **Composition (% dry mass)** |  | **Harris** | | **Samsing** | | | |
| --- | --- | --- | --- | --- | --- | --- | --- |
|  | **Species** | 2018 Summer | 2020 Summer | 2018 Summer | 2019 Winter | 2019 Summer | 2020 Summer |
| **Carbon** | *Macrocystis pyrifera* | 29.30 ± 1.90 |  | 28.70 ± 1.19 | 28.22 ± 2.31 | 32 ± 1.72 |  |
|  | *Hedophyllum nigripes* | 37.10 ± 1.51 |  | 33.10 ± 1.41 | 29.18 ± 1.13 | 38.08 ± 0.21 | 30.44 ± 0.81 |
|  | *Neoagarum fimbriatum* | 33.18 ± 0.44 |  | 32.02 ± 0.49 | 32.80 ± 0.32 | 33.78 ± 0.78 | 32.38 ± 0.43 |
|  | *Agarum clathratum* |  | 30.14 ± 1.44 |  |  |  | 36.50 ± 1.41 |
|  | *Laminaria setchellii* |  |  |  |  |  | 32.02 ± 0.96 |
|  | *Pleurophycus gardneri* |  |  |  |  |  | 31.60 ± 0.57 |
| **Nitrogen** | *Macrocystis pyrifera* | 0.56 ± 0.25 |  | 1.04 ± 0.15 | 1.6 ± 0.29 | 1.12 ± 0.39 |  |
|  | *Hedophyllum nigripes* | 0.74 ± 0.02 |  | 1.16 ± 0.07 | 2.56 ± 0.09 | 1.24 ± 0.08 | 1.32 ± 0.06 |
|  | *Neoagarum fimbriatum* | 1.46 ± 0.04 |  | 1.66 ± 0.08 | 2.28 ± 0.10 | 1.94 ± 0.07 | 1.90 ± 0.10 |
|  | *Agarum clathratum* |  | 1.8 ± 0.12 |  |  |  | 2.34 ± 0.24 |
|  | *Laminaria setchellii* |  |  |  |  |  | 0.82 ± 0.04 |
|  | *Pleurophycus gardneri* |  |  |  |  |  | 0.90 ± 0.06 |
