# Supplementary material for: Standing Crop, Turnover, and Production Dynamics of Macrocystis pyrifera and Understory Species Hedophyllum nigripes and Neoagarum fimbriatum in High Latitude Giant Kelp Forests
Source: J Phycol. 2022 Nov 17;58(6):773–88. doi: 10.1111/jpy.13291 (PMC10100489; doi:10.1111/jpy.13291)
Supplement: Supplementary file 10 — Table S6. Summary statistics for analysis of variance of macroalgal tissue nitrogen concentrations at Samsing Pinnacle Formula: nitrogen (as % dry mass) ~ season * species. [file JPY-58-773-s003.docx]

Table S6. Summary statistics for analysis of variance of macroalgal tissue nitrogen concentrations at Samsing Pinnacle

Formula: nitrogen (as % dry mass) ~ season * species

i*. Main effects*

| **Source** | **df** | **SS** | **MSE** | **F value** | **Pr(>F)** |
| --- | --- | --- | --- | --- | --- |
| season | 1 | 6.188 | 6.188 | 116.26 | <0.001 |
| species | 2 | 3.767 | 1.884 | 35.380 | <0.001 |
| season:species | 2 | 1.646 | 0.823 | 15.460 | <0.001 |
| residuals | 39 | 2.076 | 0.053 |  |  |

ii. *Tukey’s post-hoc tests for the effect of species on %N*

| **Condition1** | **Condition2** | **Mean diff** | **95% CI lower** | **95% CI upper** | **P_tukey_** |
| --- | --- | --- | --- | --- | --- |
| *H. nigripes* | *N. fimbriatum* | -0.307 | -0.512 | -0.101 | 0.002 |
|  | *M. pyrifera* | 0.400 | 0.195 | 0.605 | <0.001 |
| *M. pyrifera* | *N. fimbriatum* | -0.707 | -0.912 | -0.501 | <0.001 |

ii. *Tukey’s post-hoc tests for the effect of the interaction between season and species on %N*

| **Condition1** | **Condition2** | **Mean diff** | **95% CI lower** | **95% CI upper** | **P_tukey_** |
| --- | --- | --- | --- | --- | --- |
| Winter:*N. fimbriatum*  Summer:*M. pyrifera* | Summer:*N. fimbriatum* | 0.480 | 0.101 | 0.859 | 0.006 |
|  | Summer:*N. fimbriatum* | -0.720 | -1.029 | -0.411 | <0.001 |
| Winter:*M. pyrifera* | Summer:*N. fimbriatum* | -0.200 | -0.579 | 0.179 | 0.614 |
| Summer:*H. nigripes* | Summer:*N. fimbriatum* | -0.600 | -0.909 | -0.291 | <0.001 |
| Winter:*H. nigripes* | Summer:*N. fimbriatum* | 0.760 | 0.381 | 1.139 | <0.001 |
| Summer:*M. pyrifera* | Winter:*N. fimbriatum* | -1.200 | -1.579 | -0.821 | <0.001 |
| Winter:*M. pyrifera* | Winter:*N. fimbriatum* | -0.680 | -1.117 | -0.243 | <0.001 |
| Summer:*H. nigripes* | Winter:*N. fimbriatum* | -1.080 | -1.459 | -0.701 | <0.001 |
| Winter:*H. nigripes* | Winter:*N. fimbriatum* | 0.280 | -0.157 | 0.717 | 0.406 |
| Winter:*M. pyrifera* | Summer:*M. pyrifera* | 0.520 | 0.141 | 0.899 | 0.002 |
| Summer:*H. nigripes* | Summer:*M. pyrifera* | 0.120 | -0.189 | 0.429 | 0.851 |
| Winter:*H. nigripes* | Summer:*M. pyrifera* | 1.480 | 1.101 | 1.859 | <0.001 |
| Summer:*H. nigripes* | Winter:*M. pyrifera* | -0.400 | -0.779 | -0.021 | 0.033 |
| Winter:*H. nigripes* | Winter:*M. pyrifera* | 0.960 | 0.523 | 1.397 | <0.001 |
| Winter:*H. nigripes* | Summer:*H. nigripes* | 1.360 | 0.981 | 1.739 | <0.001 |
