# Supplementary material for: Standing Crop, Turnover, and Production Dynamics of Macrocystis pyrifera and Understory Species Hedophyllum nigripes and Neoagarum fimbriatum in High Latitude Giant Kelp Forests
Source: J Phycol. 2022 Nov 17;58(6):773–88. doi: 10.1111/jpy.13291 (PMC10100489; doi:10.1111/jpy.13291)
Supplement: Supplementary file 11 — Table S7. Summary statistics for analysis of variance of macroalgal tissue carbon concentrations at Samsing Pinnacle Formula: carbon (as % dry mass) ~ season * species. [file JPY-58-773-s007.docx]

Table S7. Summary statistics for analysis of variance of macroalgal tissue carbon concentrations at Samsing Pinnacle

Formula: carbon (as % dry mass) ~ season * species

i*. Main effects*

| **Source** | **df** | **SS** | **MSE** | **F value** | **Pr(>F)** |
| --- | --- | --- | --- | --- | --- |
| season | 1 | 82.940 | 82.940 | 14.638 | <0.001 |
| species | 2 | 126.490 | 63.240 | 11.161 | <0.001 |
| season:species | 2 | 69.170 | 34.590 | 6.104 | 0.005 |
| residuals | 39 | 220.990 | 5.670 |  |  |

ii. *Tukey’s post-hoc tests for the effect of species on %N*

| **Condition1** | **Condition2** | **Mean diff** | **95% CI lower** | **95% CI upper** | **P_tukey_** |
| --- | --- | --- | --- | --- | --- |
| *H. nigripes* | *N. fimbriatum* | -3.227 | -5.344 | -1.109 | 0.002 |
|  | *M. pyrifera* | 0.587 | -1.531 | 2.704 | 0.779 |
| *M. pyrifera* | *N. fimbriatum* | 3.813 | 1.696 | 5.931 | <0.001 |

ii. *Tukey’s post-hoc tests for the effect of the interaction between season and species on %N*

| **Condition1** | **Condition2** | **Mean diff** | **95% CI lower** | **95% CI upper** | **P_tukey_** |
| --- | --- | --- | --- | --- | --- |
| Winter:*N. fimbriatum*  Summer:*M. pyrifera* | Summer:*N. fimbriatum* | -0.100 | -4.006 | 3.806 | 1.000 |
|  | Summer:*N. fimbriatum* | -2.550 | -5.739 | 0.639 | 0.183 |
| Winter:*M. pyrifera* | Summer:*N. fimbriatum* | -4.680 | -8.586 | -0.774 | 0.011 |
| Summer:*H. nigripes* | Summer:*N. fimbriatum* | 2.690 | -0.499 | 5.879 | 0.141 |
| Winter:*H. nigripes* | Summer:*N. fimbriatum* | -3.720 | -7.626 | 0.186 | 0.070 |
| Summer:*M. pyrifera* | Winter:*N. fimbriatum* | -2.450 | -6.356 | 1.456 | 0.430 |
| Winter:*M. pyrifera* | Winter:*N. fimbriatum* | -4.580 | -9.090 | -0.070 | 0.045 |
| Summer:*H. nigripes* | Winter:*N. fimbriatum* | 2.790 | -1.116 | 6.696 | 0.289 |
| Winter:*H. nigripes* | Winter:*N. fimbriatum* | -3.620 | -8.130 | 0.890 | 0.180 |
| Winter:*M. pyrifera* | Summer:*M. pyrifera* | -2.130 | -6.036 | 1.776 | 0.582 |
| Summer:*H. nigripes* | Summer:*M. pyrifera* | 5.240 | 2.051 | 8.429 | <0.001 |
| Winter:*H. nigripes* | Summer:*M. pyrifera* | -1.170 | -5.076 | 2.736 | 0.945 |
| Summer:*H. nigripes* | Winter:*M. pyrifera* | 7.370 | 3.464 | 11.276 | <0.001 |
| Winter:*H. nigripes* | Winter:*M. pyrifera* | 0.960 | -3.550 | 5.470 | 0.987 |
| Winter:*H. nigripes* | Summer:*H. nigripes* | -6.410 | -10.316 | -2.504 | <0.001 |
